# On the Stability of Rotating Drops

**DOI:** 10.6028/jres.120.007

**Published:** 2015-04-20

**Authors:** A. K. Nurse, S. R. Coriell, G. B. McFadden

**Affiliations:** National Institute of Standards and Technology, Gaithersburg, MD 20899

**Keywords:** bifurcation, linear stability, rotating drop, toroids, variational principle

## Abstract

We consider the equilibrium and stability of rotating axisymmetric fluid drops by appealing to a variational principle that characterizes the equilibria as stationary states of a functional containing surface energy and rotational energy contributions, augmented by a volume constraint. The linear stability of a drop is determined by solving the eigenvalue problem associated with the second variation of the energy functional. We compute equilibria corresponding to both oblate and prolate shapes, as well as toroidal shapes, and track their evolution with rotation rate. The stability results are obtained for two cases: (i) a prescribed rotational rate of the system (“driven drops”), or (ii) a prescribed angular momentum (“isolated drops”). For families of axisymmetric drops instabilities may occur for either axisymmetric or non-axisymmetric perturbations; the latter correspond to bifurcation points where non-axisymmetric shapes are possible. We employ an angle-arc length formulation of the problem which allows the computation of equilibrium shapes that are not single-valued in spherical coordinates. We are able to illustrate the transition from spheroidal drops with a strong indentation on the rotation axis to toroidal drops that do not extend to the rotation axis. Toroidal drops with a large aspect ratio (major radius to minor radius) are subject to azimuthal instabilities with higher mode numbers that are analogous to the Rayleigh instability of a cylindrical interface. Prolate spheroidal shapes occur if a drop of lower density rotates within a denser medium; these drops appear to be linearly stable. This work is motivated by recent investigations of toroidal tissue clusters that are observed to climb conical obstacles after self-assembly [Nurse et al., Journal of Applied Mechanics 79 (2012) 051013].

## 1. Introduction

Analyses of the dynamics of a rotating liquid drop held together by surface tension were initiated by Plateau [1]. In his work a liquid drop was immersed in an immiscible liquid which has about the same density but a much smaller viscosity than the drop. The drop was then spun at a controllable rate using a rotating rod that threaded the drop axis. Spinning of the drop produces significant deviations from the spherical equilibrium shape that is obtained for stationary drops. By matching the density of the drop and its surrounding medium, gravitational effects can be minimized in a terrestrial experiment. Assuming rigid body motion and taking into account solely axisymmetric drop configurations, the drops evolve from spherical configurations at zero rotation rate through a family of axisymmetric shapes that progressively flatten at the poles while developing an equatorial bulge. At large enough rotation rates, Plateau observed transient toroidal configurations that tended to break up into smaller drops (see also [2]).

Studies of rotating drops with significant density contrasts have also been performed in microgravity environments [3]. Due to the qualitative similarity between Plateau’s rotating liquid masses held together by surface tension and self-gravitating celestial bodies, there have been many theoretical studies of the resulting equilibrium configurations and their stability, including work by Rayleigh [4], Maclaurin [5], Lyttleton [6], Chandrasekhar [7], Ross [8] and Brown and Scriven [9], and Myshkis et al. [10].

Recently there has been interest in the stability of toroidal shapes. Experimentally, macroscopic liquid toroidal droplets [11] and nanoscale toroids of varying sizes have been carefully generated and observed [12, 13]. Analysis indicates that the stability of these toroidal shapes is related to the aspect ratio of the major and minor radii. Both groups report that toroids with a small aspect ratio tend to coalesce to form a single spherical droplet, while thinner toroids, i.e., those with large aspect ratio, mainly break up into smaller droplets.

Experiments on neonatal fibroblast cells that have self assembled into a toroidal cluster about the base of a conical pillar, have shown that the toroidal cluster will actively do work to climb the pillar to become a sphere or will remain at the base of the pillar and break up to form smaller clusters [14]. Subsequent theoretical work on this self assembled system points to the surface energy as the configurational driving force for the climbing motion of the cluster [15]. This suggests that the fate of the cluster is determined by its size. As such, thinner toroidal clusters do not climb the conical pillar and the development of localized deformations along its circumference is considered to be a result of the unstable growth of surface fluctuations.

These studies have revived interest in the stability of a rotating toroidal drop held together by surface tension. In this paper the stationary points of an energy functional are used to determine the equilibrium shapes of spheroidal and toroidal drops. The stability of the drops is then determined by examining the second variation of their energy functionals. The loss of stability of an equilibrium drop can indicate a transition to another equilibrium shape or signal the impending loss of the existence of the equilibrium drop itself.

The energy functional that we employ takes the form of a Lagrangian representing the difference between a drop’s potential energy, due to capillary forces, and the kinetic energy of rotation, subject to a volume constraint. This formulation is analogous in many ways to that for the classical problem for the equilibrium and stability of rotating, self-gravitating drops, where the potential energy is then due to a Newtonian gravitational potential [6, 16]. In that case there is a class of equilibria given by axisymmetric ellipsoids that take the form of oblate spheroids (shaped like “hamburger buns” with respect to the rotational axis). In our case there also are axisymmetric solutions that resemble oblate ellipsoids. These occur when the density of the drop exceeds that of the surrounding medium, so that due to centripetal acceleration the drop tends to bulge at the equator and flatten at the pole. On the other hand if the density of the surrounding medium exceeds that of the drop a family of solutions that instead resemble prolate ellipsoids (“cigar-shaped” with respect to the rotational axis) are possible. We will refer to these families of solutions as oblate spheroids and prolate spheroids, respectively, with the understanding that in our case these solutions are not literally axisymmetric ellipsoids of revolution. More generally we will refer to our solutions as spheroidal or toroidal depending on their topology.

For the drop undergoing rigid body rotation with a fluid of a different density, the application of an external torque may be necessary to maintain a given rotation rate as the moment of inertia of the drop changes due to variations in the shape of the drop. Here, such a drop is referred to as a driven drop. Alternatively, if there is no applied external torque, the angular momentum of the drop is conserved. If the drop rotates with constant angular momentum, it is termed an isolated drop. The energy functional for the driven drop is a function of the drop’s shape and the rate of rotation, while the energy functional of the isolated drop is a function of the drop’s shape and its angular momentum. This latter energy functional is formulated by a Legendre transform of the former functional to account for the constant angular momentum constraint, forming what is termed the Routhian in classical mechanics [17].

We first describe the variational formulation of the problem, including the Euler equation governing the equilibrium drops that results from the first variation of the energy functional. In §3 we discuss the second variation that governs the stability of the equilibrium drops. Our numerical techniques are described in §4, followed by numerical results in §5. Some conclusions are given in §6.

## 2. Variational Formulation

We describe the variational formulation for the equilibrium and stability of rotating spheroidal and toroidal drops. The case of a rigidly rotating system consisting of a drop confined by a co-rotating surrounding medium is considered first, followed by the modifications necessary to treat the rotation of an isolated drop that conserves its angular momentum. An example of the former would be a two-fluid system in a container that is attached to a platter revolving at a constant rate. An example of the latter would be a isolated drop rotating in vacuum in a microgravity environment.

### 2.1 Forced Rotation at a Prescribed Rate

**Fig. 1:**
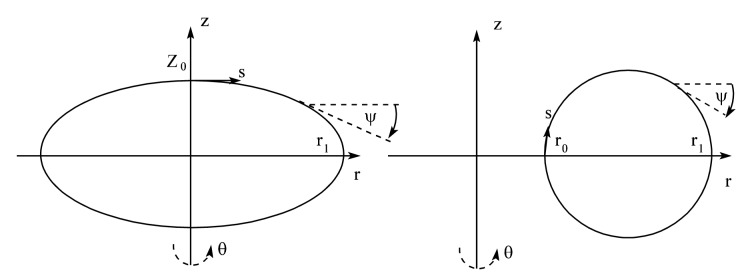
Schematic diagrams showing cross-sections of a rotating spheroidal drop (left) and a rotating toroidal drop (right). Here the arclength *s* increases in the clockwise direction, and the tangent angle *ψ* is measured with respect to the horizontal.

The equilibrium of a driven axisymmetric drop that undergoes rigid body rotation at a specified angular velocity in tandem with a surrounding co-rotating medium can be described in terms of the stationary points of an energy functional that includes contributions from the kinetic energy and surface energy of the system. For simplicity we begin by formulating the variational principle in a cylindrical coordinate system in which the drop surface has the form *z* = *f*(*r*) for *r*_0_ < *r < r*_1_ and *z >* 0 ; we will assume that the drops have a mid-plane of symmetry about *z* = 0. We later generalize to a body-fitted set of coordinates employing angle/arclength variables that avoids difficulties associated with infinite slopes and is more suitable for the stability determination of distorted drops. For a spheroidal drop *r*_0_ = 0 corresponds to the axis of rotation and *r*_1_ is the equatorial radius, with a vanishing slope *f_r_* = *df/dr* at *r* = 0 and a tangent angle *ψ*, defined by tan *ψ* = *f_r_*, of *ψ* = *−π*/2 at *r* = *r*_1_ where *f*(*r*_1_) = 0. The polar radius of the drop is *Z*_0_ = *f*(0). For a toroidal drop *r*_0_ > 0 is the inner radius of the toroid and *r*_1_ *> r*_0_ is the outer radius, with a tangent angle of *ψ* = *π*/2 at *r* = *r_0_* where *f*(*r*_0_) = 0, and *ψ* = *−π*/2 at *r* = *r*_1_ where *f*(*r*_1_) = 0. Schematic diagrams are shown in Fig. 1.

The effective energy functional is written as

$$ℰ[f,\Omega ]=\gamma A[f]-\frac{1}{2}\Omega^{2}ℐ[f]-PV[f],$$ (1)

where *γ* is the surface energy of the drop, Ω is the given rotation rate, and

$$A[f]=4\pi \int_{r_{0}}^{r_{1}}r\sqrt{1+f_{r}^{2}}dr$$ (2)

is the total surface area of the drop. The effective kinetic energy of the system is Ω^2^ℐ/2, where

$$ℐ[f]=4\pi \Delta \rho \int_{r_{0}}^{r_{1}}r^{3}f(r)dr$$ (3)

is the moment of inertia. The total volume of the drop is

$$V[f]=4\pi \int_{r_{0}}^{r_{1}}rf(r)dr,$$ (4)

and *P* is a Lagrange multiplier that is used to enforce a constraint $V\left[f\right]=V_{0}$ of constant volume $V_{0}$. Here Δ*ρ* = *ρ*_inner_ − *ρ*_outer_ *is* the difference between the drop density *ρ*_inner_ and the density of the exterior medium *ρ*_outer_. Rotation in a vacuum or medium of negligible density then corresponds to Δ*ρ* = *ρ*_inner_ > 0, whereas a drop in a heavier surrounding fluid medium with density *ρ*_outer_ > *ρ*_inner_ would correspond to a negative density difference Δ*ρ <* 0. We are assuming there are no gravitational effects.

Equilibrium of the drop is then described by requiring the energy functional to be stationary to perturbations *δf* in the shape that conserve the volume and satisfy appropriate boundary conditions at *r* = *r*_0_ and *r* = *r*_1_,

$$0=\delta ℰ=\gamma \delta A[f]-\frac{1}{2}\Delta \rho \Omega^{2}\delta ℐ[f]-P\delta V[f],$$ (5)

which leads to the Euler equation

$$-\frac{\gamma }{r}\frac{d}{dr}\left[r\frac{f_{r}}{\sqrt{1+f_{r}^{2}}}\right]=P+\frac{\Delta \rho \Omega^{2}}{2}r^{2},$$ (6)

for the shape *f* (*r*) and the Lagrange multiplier *P*, which is chosen so that the volume constraint

$$4\pi \int_{r_{0}}^{r_{1}}rf(r)dr=V_{0}$$ (7)

is satisfied. The Euler equation is equivalent to the Laplace-Young boundary condition *γK* = *p*_inner_ − *p*_outer_ at a fluid-fluid interface, where *K* is the mean curvature of the interface and *p*_inner_ and *p*_outer_ are the local pressures on the inside and outside of the drop, respectively. In a hydrodynamic description of the motion based on the Navier-Stokes equations [18], in each phase the pressure satisfies a radial momentum balance *ρr*Ω^2^ = *dp/dr* for a rigid body motion *r*Ω in the azimuthal direction, which integrates to $p–\rho \Omega^{2}r^{2}/2=\bar{p}=\text{constant}$. The Lagrange multiplier is then given by the difference between the constants of integration ${\bar{p}}_{\text{inner}}-{\bar{p}}_{\text{outer}}$. For a spheroidal drop this difference corresponds to the jump in pressure across the surface at the axis of rotation *r* = 0.

The Euler equation has an explicit first integral

$$-\gamma \frac{f_{r}}{\sqrt{1+f_{r}^{2}}}=\frac{Pr}{2}+\frac{\Delta \rho \Omega^{2}r^{3}}{8}+\frac{C}{r},$$ (8)

where *C* is a constant of integration. The first integral can be solved for *f_r_* to reduce the solution to quadrature. For spheroidal solutions with *f_r_*(0) = 0 the integration constant *C* vanishes. The oblate spheroidal solutions that result if Δ*ρ >* 0 were obtained in terms of elliptic integrals by Chandrasekhar [7]. The prolate solutions that result if Δ*ρ <* 0 can also be described in terms of elliptic integrals; these solutions are summarized in Appendix 7.1. In the toroidal case, *f_r_* tends to positive infinity at the inner radius *r*_0_ and the integration constant satisfies

$$-\gamma =\frac{{Pr}_{0}}{2}+\frac{\Delta \rho \Omega^{2}r_{0}^{3}}{8}+\frac{C}{r_{0}}.$$ (9)

There is an analogous expression relating *C* and the outer radius *r*_1_ where *f_r_* tends to negative infinity. The existence of toroidal solutions was proved in 1984 by Gulliver [19]. The spheroidal and toroidal solutions will be described in more detail below when we discuss the stability results in § 5.

### 2.2 Free Rotation of an Isolated Drop

If an isolated drop is freely rotating rather than being driven by an external torque that provides a constant rotation rate, it is appropriate to formulate the problem in terms of the drop’s angular momentum *L*, which is conserved by the motion. With the explicit form for the energy functional ℰ[*f*, Ω] given in Eq. (1), the angular momentum functional *L*[*f*, Ω] is given formally by

$$L[f,\Omega ]=-\frac{\partial }{\partial \Omega }ℰ[f,\Omega ]=\Omega ℐ[f].$$ (10)

We define the Routhian functional ℛ[*f, L*] (see, e.g., [6, 9]) via a Legendre transformation [20] with respect to Ω,

$$ℛ[f,L]=ℰ[f,\Omega ]-\Omega \frac{\partial }{\partial \Omega }ℰ[f,\Omega ],$$ (11)

where in the right hand side the rotation rate is now regarded as a functional Ω[*f*, *L*] = *L/*ℐ[*f*] that is obtained by inversion of the relation (10). This leads to the expression

$$ℛ[f,L]=\gamma A[f]+\frac{L^{2}}{2ℐ[f]}-PV[f],$$ (12)

whose first variation, taken at constant *L*,

$$\delta ℛ=\gamma \delta A-\frac{L^{2}}{2{ℐ}^{2}}\delta ℐ-P\delta V,$$ (13)

leads to the same Euler equation (6) as for the driven drop, since we have *L* = ℐΩ in each case. Thus the equilibrium states for the driven drops and the isolated drops are the same, although their stability differs, as we describe next.

## 3. Second Variation

We next describe the stability of the drops in terms of the second variation of their energy functionals. The equations for the second variation involve the equilibrium shape and its spatial derivatives, and it is convenient to first re-express the unperturbed shape in terms of angle/arclength variables to obtain a more tractable version of the stability equations.

### 3.1 Angle/Arclength Coordinates on the Drop

The axisymmetric equilibrium shapes can be parametrized in terms of their arclength *s* as *r* = *R*(*s*) and *z* = *Z*(*s*), where *s* = 0 is taken to correspond to the point *r* = *r*_0_. Their derivatives are given by *R_s_* = cos*ψ*(*s*) and *Z_s_* = sin*ψ*(*s*) for 0 < *s* < *S_T_*, where *ψ* is the local tangent angle to the shape and *S_T_* is the total arclength of the upper half (*z* > 0) of the shape. The Euler equation (6) is then

$$-\gamma \psi_{s}=\gamma \frac{\sin\psi }{R}+P+\frac{\Delta \rho \Omega^{2}}{2}R^{2},$$ (14)

where the mean curvature is given by *K* =*ψ_s_* + sin(*ψ*)/*r*. We note that the first integral (8) can be written in the form

$$-\gamma \sin\psi =\frac{PR}{2}+\frac{\Delta \rho \Omega^{2}}{8}R^{3}+\frac{C}{R}.$$ (15)

In the spheroidal case with *C* = 0 this expression can be used to eliminate the singular term sin(*ψ*)/*R* in Eq. (14) to give the alternate expression

$$-\gamma \psi_{s}=\frac{P}{2}+\frac{3\Delta \rho \Omega^{2}}{8}R^{2},$$ (16)

which is regular at *s* = 0 where *R*(0) = 0. On the other hand, since for the toroidal drop *R*(0) = *r*_0_ > 0 this singularity does not arise in that case and Eq. (14) can be used as written. In either case the total volume is given by

$$V=4\pi \int_{0}^{S_{T}}R(s)Z(s)\cos\psi (s)ds.$$ (17)

### 3.2 Body-Fitted Coordinate System

To compute the second variation we employ a body-fitted coordinate system (*s*,*θ*,*w*) where *s* is arclength, *θ* is the azimuthal angle about the rotation axis, and *w* is distance measured along the local outward normal to the drop surface. The mapping from (*s*,*θ*,*w*) to cylindrical coordinates (*r*,*θ*,*z*) is then given by

r=R(s)−wsinψ(s),z=Z(s)+wcosψ(s), (18)

where the outward normal has components (*n_r_*, *n_z_*) = (−sin*ψ*, cos*ψ*) in the *r* – *z* plane. These coordinates are well-defined in a neighborhood of the drop’s surface for small enough values of |*w*|. These local coordinates are orthogonal with the line element

$$d\ell^{2}={\left[1-w\psi_{s}^{2}\right]}^{2}ds^{2}+{\left[R-w\sin\psi \right]}^{2}d\theta^{2}+dw^{2},$$ (19)

and the volume element $dV=[1-w\psi_{s}^{2}][R-w\sin\psi ]dsd\theta dw$. A perturbation to the drop can be written in terms of a normal displacement given by a function *w* = *W*(*s, θ*) along the normal direction, with the unperturbed drop corresponding to the surface *w* = 0. The perturbation satisfies Neumann boundary conditions *W_s_*(0,*θ*) = *W_s_* (*S_T_, θ*) = 0.

To compute the first and second variations of the energy functional, we formally expand the interface displacement in terms of a small parameter *ε*,

$$W(s,\theta ,\varepsilon )=\varepsilon W^{\left(1\right)}(s,\theta )+\frac{1}{2}\varepsilon^{2}W^{\left(2\right)}(s,\theta )+\ldots$$ (20)

The various functionals can written in terms of *W*(*s, θ, ε*), e.g., ℱ[*W*], and similarly expanded as ℱ[*W*] = *ε*ℱ^(1)^ *+*(*ε*^2^/2)ℱ^(2)^ +…, giving the results

$$V^{\left(1\right)}=2\int_{0}^{2\pi }\int_{0}^{S_{T}}RW^{\left(1\right)}d\theta ds,$$ (21)

$$V^{\left(2\right)}=2\int_{0}^{2\pi }\int_{0}^{S_{T}}\left\{RW^{\left(2\right)}-(R\psi_{s}+\sin\psi ){\left[W^{\left(1\right)}\right]}^{2}\right\}d\theta ds,$$ (22)

$${ℐ}^{\left(1\right)}=2\Delta \rho \int_{0}^{2\pi }\int_{0}^{S_{T}}R^{3}W^{\left(1\right)}d\theta ds,$$ (23)

$${ℐ}^{\left(2\right)}=2\Delta \rho \int_{0}^{2_{\pi }}\int_{0}^{S_{T}}\{R^{3}W^{\left(2\right)}-(R^{3}\psi_{s}+3R^{2}\sin\psi ){\left[W^{\left(1\right)}\right]}^{2}\}d\theta ds,$$ (24)

$$A^{\left(1\right)}=-2\int_{0}^{2\pi }\int_{0}^{S_{T}}W^{\left(1\right)}(R\psi_{s}+\sin\psi )d\theta ds,$$ (25)

$$A^{\left(2\right)}=2\int_{0}^{2\pi }\int_{0}^{S_{T}}\left\{2\psi_{s}\sin\psi {\left[W^{\left(1\right)}\right]}^{2}+R{\left[W_{s}^{\left(1\right)}\right]}^{2}+\frac{1}{R}{\left[W_{\theta }^{\left(1\right)}\right]}^{2}-(R\psi_{s}+\sin\psi )W^{\left(2\right)}\right\}d\theta ds.$$ (26)

### 3.3 Driven Drop

The first variation of the energy functional for the driven drop is then ${ℰ}^{\left(1\right)}=\gamma A^{\left(1\right)}-\Omega^{2}/2{ℐ}^{\left(1\right)}-PV^{\left(1\right)}$, or

$${ℰ}^{\left(1\right)}=2\int_{0}^{2\pi }\int_{0}^{S_{T}}\left\{-\gamma (R\psi_{s}+\sin\psi )-\frac{\Delta \rho \Omega^{2}}{2}R^{3}-PR\right\}W^{\left(1\right)}d\theta ds,$$ (27)

and requiring ℰ^(1)^ =0 for arbitrary variations *W*^(1)^ recovers the Euler equation (14). Similarly, the second variation ℰ^(2)^ is given by

$$\begin{array}{c} {ℰ}^{\left(2\right)}=2\int_{0}^{2\pi }\int_{0}^{S_{T}}\{\left[2\gamma \psi_{s}\sin\psi +\frac{\Delta \rho \Omega^{2}}{2}[R^{3}\psi_{s}+3R^{2}\sin\psi ]+P(R\psi_{s}+\sin\psi )\right]{\left[W^{\left(1\right)}\right]}^{2}\\ +[\gamma R]{\left[W_{s}^{\left(1\right)}\right]}^{2}+[\frac{\gamma }{R}]{\left[W_{\theta }^{\left(1\right)}\right]}^{2}\}dsd\theta \\ -2\int_{0}^{2\pi }\int_{0}^{S_{T}}\left\{\left[\gamma (R\psi_{s}+\sin\psi )+\frac{\Delta \rho \Omega^{2}}{2}R^{2}+PR\right]W^{\left(2\right)}\right\}dsd\theta . \end{array}$$ (28)

The latter integrand proportional to *W*^(2)^ vanishes because of the Euler equation, so that ℰ^(2)^ is a quadratic functional in the variation *W*^(1)^. The stability of the system is then determined by the sign of the second variation. This sign is most easily determined by diagonalizing the quadratic form, subject to the constraint

$$0=V^{\left(1\right)}=2\int_{0}^{2\pi }\int_{0}^{S_{T}}RW^{\left(1\right)}d\theta ds.$$ (29)

The diagonalization of the quadratic form is equivalent to an eigenvalue problem, which leads to the Sturm-Liouville equation

$$\left\{-\gamma \psi_{s}^{2}-\gamma \frac{\sin^{2}\psi }{R^{2}}+\Delta \rho \Omega^{2}R^{2}\frac{\sin\psi }{R}\right\}W^{\left(1\right)}-\frac{\gamma }{R}\frac{\partial }{\partial s}[RW_{s}^{\left(1\right)}]-\frac{\gamma }{R^{2}}\frac{\partial^{2}}{\partial \theta^{2}}W^{\left(1\right)}=\gamma W^{\left(1\right)}+\mu ,$$ (30)

where *λ* is the eigenvalue and *μ* is a Lagrange multiplier for the volume constraint in Eq. (29), which must also be enforced. Here we have again used the Euler equation to simplify the final expression; note that *P* is absent from this equation. Expanding a general perturbation in terms of the orthonormal eigenmodes (*W_j_*(*s,θ*),* λ_j_*) of the Sturm-Liouville equation,

$$W^{\left(1\right)}(s,\theta )=\sum_{j}a_{j}W_{j}(s,\theta ),$$ (31)

then produces the diagonalized expression

$${ℰ}^{\left(2\right)}=2\sum_{j}\lambda_{j}|a_{j}{|}^{2}\int_{0}^{2\pi }\int_{0}^{S_{T}}R{\left[W_{j}(s,\theta )\right]}^{2}d\theta ds.$$ (32)

Here *a_j_* is the expansion coefficient of *W*^(1)^ with respect to the eigenmode *W_j_*,

$$a_{j}=\int_{0}^{2\pi }\int_{0}^{S_{T}}RW^{\left(1\right)}(s,\theta )W_{j}(s,\theta )d\theta ds.$$

Stability of the driven drop is obtained, viz. ℰ^(2)^ *>* 0, if *λ_j_* is positive for all eigenmodes, and instability occurs if any eigenvalue *λ_j_* is negative.

### 3.4 Isolated Drop

For the isolated drop, the expansion of the Routhian proceeds in a similar fashion, leading to the second variation

$${ℛ}^{\left(2\right)}=\gamma A^{\left(2\right)}-\frac{L^{2}}{2{ℐ}^{2}}{ℐ}^{\left(2\right)}-PV^{\left(2\right)}+\frac{L^{2}}{{ℐ}^{3}}{\left[{ℐ}^{\left(1\right)}\right]}^{2},$$ (33)

which differs from the expression for ℰ^(2)^ by the latter positive term proportional to [ℐ^(1)^]^2^; here ℐ= *L*/Ω. is the unperturbed moment of inertia. In this case diagonalizing ℛ^(2)^ leads to an integro-differential eigenvalue problem,

$$\begin{array}{l} \left\{-\gamma \psi_{s}^{2}-\gamma \frac{\sin^{2}\psi }{R^{2}}+\Delta \rho \Omega^{2}R^{2}\frac{\sin\psi }{R}\right\}W^{\left(1\right)}-\frac{\gamma }{R}\frac{\partial }{\partial s}[RW_{s}^{\left(1\right)}]-\frac{\gamma }{R^{2}}\frac{\partial^{2}}{\partial \theta^{2}}W^{\left(1\right)}\\ \;+2(\Delta \rho {)}^{2}\left(\frac{L^{2}}{{ℐ}^{3}}\right)[R(s){]}^{2}\int_{0}^{2\pi }\int_{0}^{S_{T}}{\left[R(s^{\prime })\right]}^{3}W^{\left(1\right)}(s^{\prime },\theta )d\theta ds^{\prime }=\lambda W^{\left(1\right)}+\mu . \end{array}$$ (34)

The isolated drop is stable if the eigenvalues *λ_j_* are positive for all eigenfunctions of Eq. (34).

Since the coefficients of both Eqs. (30) and (34) are independent of the azimuthal angle *θ*, normal modes of the form *W*^(1)^(*s,9*) = *w*^(1)^(*s*)*cosnθ* are solutions, which reduces Eq. (30) to an ordinary differential equation (ODE) for the corresponding eigenmodes *w*^(1)^ (*s*). The non-axisymmetric modes with *n* ≠ 0 then automatically satisfy the volume constraint since the integrals over *θ* vanish, and the associated Lagrange multiplier *μ* can be taken to vanish. The volume constraint must still be applied for the axisymmetric modes with *n* = 0. Similarly Eq. (34) becomes an integro-ordinary-differential equation that must be solved along with a volume constraint for axisymmetric modes. The equation for non-axisymmetric modes with *n* ≠ 0 also reduces to an unconstrained ODE, and the stability problem for non-axisymmetric modes are identical for driven and isolated drops.

The normal mode solutions can generally be divided into families that are even or odd about a mid-plane of symmetry at *z* = 0. As discussed by Brown and Scriven [9], for the related cases of rotating drops that are held together by self-gravitation it is known that the drops have reflective symmetry about their equatorial plane [21]. Brown and Scriven therefore confined their finite element computations to solutions with even symmetry about *z* = *0.* We have computed normal modes with either even or odd symmetry, and have found instabilities only for modes with reflective symmetry about *z* = 0. There are also neutrally-stable modes with odd symmetry about the equatorial plane that correspond to simple energy-preserving translations along the *z* -axis. With the exception of these translation modes, the modes with odd symmetry about *z* = 0 are found to be stable, and so we will confine our discussion to the even modes. The numerical procedures used to solve these corresponding eigenvalue problems are summarized next.

## 4. Numerical Techniques

The eigenvalue problems for determining the stability of the rotating drops are intractable analytically except in special cases, and we have resorted to numerical techniques for their solution. We have used two complementary approaches. Firstly, a finite difference discretization of the Sturm-Liouville equations can be used to produce a matrix eigenvalue problem, which produces *N* approximate eigenvalues for a system using *N* mesh points. Secondly, we have used an ODE solver in tandem with a shooting procedure to compute individual eigenmodes. The latter procedure is quite accurate provided adequate starting values are available to estimate the eigenvalues; we have used the matrix approximation to furnish the necessary initial guesses. Since the coefficients of the ODE’s involve the shape of the unperturbed drop, the Euler equations are also solved numerically to provide the appropriate values at the mesh points of the matrix formulation or at the internal integration steps of the ODE solver. Some details are provided in Appendix 7.2 and 7.3.

## 5. Numerical Results

We first consider the case of heavier drops rotating in a lighter medium, followed by the case of drops that are lighter than their surroundings.

### 5.1 Base States for Δ*ρ >*0

**Fig. 2:**
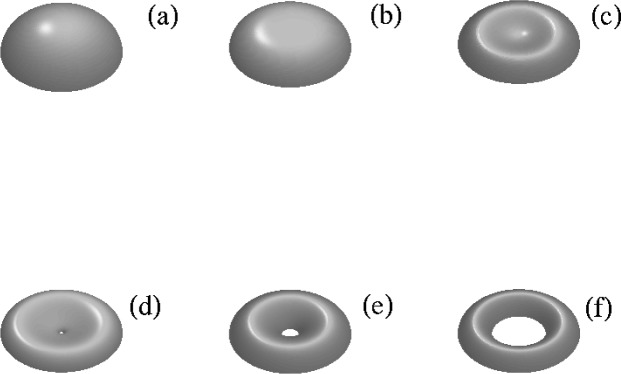
Top: Upper half of spheroidal drop shapes illustrating the development of equatorial bulge and flattening at the poles. Bottom: Upper half of toroidal drop shapes showing development of the inner hole. (a) Ω_*_, = 0.600, L_*_ = 0.340. (b) Ω_*_ = 0.707, L_*_ = 0.494. (c) Ω_*_ = 0.754, L_*_ = 0.729. (d) Ω_*_ = 0.727, L_*_ = 0.899. (e) Ω_*_ = 0.678, L_*_ = 0.699.(f) Ω_*_ = 0.484, L_*_ = 0.745.

The evolution of the axisymmetric drop shapes with Δ*ρ* > 0 as the rate of rotation Ω is varied has been described by a number of authors [7, 9]; some examples are illustrated in Fig. 2. Here we have defined the dimensionless rotation rate Ω_*_, the moment of inertia ℐ_*_, and the angular momentum L_*_ via

$$\Omega_{*}^{2}=\frac{\Delta \rho \Omega^{2}R_{0}^{3}}{8\gamma },\;{ℐ}_{*}\frac{ℐ}{4\Delta \rho R_{0}^{3}},\;L_{*}={ℐ}_{*}\Omega_{*}$$ (35)

where *R_0_* is the radius of the sphere with equivalent volume, $V_{0}=4\pi R_{0}^{3}/3$. For small rates of rotation the drops are nearly spherical, and as the rate of rotation increases the drops develop an equatorial bulge while flattening at the poles. The continual decrease in polar radius eventually produces dimpling of the surface at the pole and the drop becomes non-convex. The family of spheroidal drops terminates at a point in parameter space where the polar radius *Z_0_* of the drop vanishes and the drop pinches off at the poles. There is also a nearby family of toroidal drops which originate near this point in parameter space; in this case, the inner radius *r*_0_ of the torus tends to zero as the “hole” of the toroid closes up. The pinching of the spheroidal drops and the “healing” of the toroidal hole are illustrated in the sequences shown in Fig. 2.

**Fig. 3:**
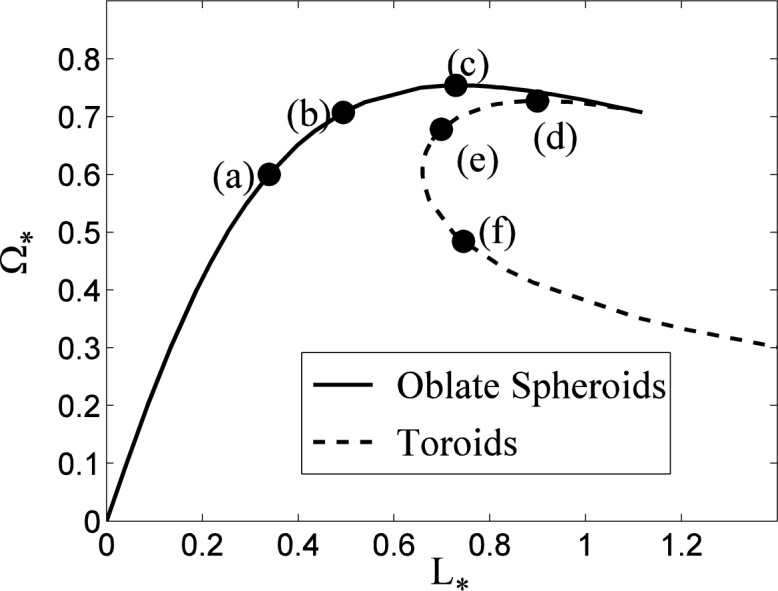
Rotation rate Ω_*_ versus angular momentum L_*_ for the family of oblate spheroidal drops and toroidal drops. Shapes shown in Fig. 2 are indicated by symbols.

The relation between angular rotation rate and angular momentum for the spheroidal and toroidal drop families is shown in Fig. 3. The angular momentum *L* of the spheroidal drops initially increases with rotation rate, but the rotation rate eventually decreases as the angular momentum continues to increase (see Fig. 3). As the inner radius of the toroids increases, the angular momentum of the drops initially decreases, then reverses and increases steadily as the cross section of the toroids becomes more and more circular. We note that the spheroidal and toroidal families do not merge with one another, although their curves are very close at their respective terminal points in Fig. 3 near L_*_ = 1.1194. The solution curve for the spheroidal family can actually be smoothly extended to include self-intersecting drops for L_*_ > 1.1194 where the polar radius has become negative [*Z*(0) < 0]. In addition, the boundary conditions differ at *s* = 0, since the spheroidal drops have a horizontal tangent there, while the toroidal drops have a vertical tangent. As a result, as we will see below, the normal modes of the spheroidal and toroidal modes are not coincident at L_*_ = 1.1194.

### 5.2 Linear Stability of the Oblate Spheroids

**Fig. 4:**
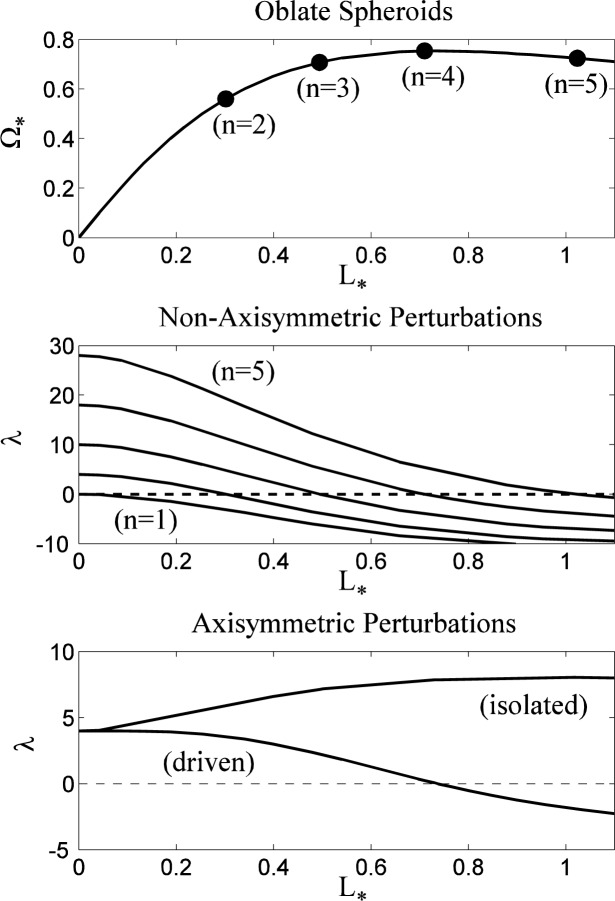
Linear stability of rotating spheroidal drops. In the upper plot the points of marginal stability to perturbations with mode number *n* are shown on the Ω_*_ − L_*_ oblate spheroidal solution branch. In the middle plot the least stable values of *λ* as a function of L_*_ are shown for *n* =1,2,3,4, and 5 (bottom to top). The drops are unstable to modes with *λ*< 0, and the crossing points where *λ*= 0 for each *n* correspond to the symbols shown in the upper plot. In the lower plot the least stable values of *λ* for axisymmetric disturbances (*n* = 0) are shown for driven and isolated drops. The driven drop is unstable to an axisymmetric perturbation at the limit point where the spheroidal family of solutions reaches its largest rotation rate.

The stability of rotating oblate spheroidal drops has been considered previously by a number of authors, including Chandrasekhar [7], Brown and Scriven [9], and Heine [22]; the latter two papers include the computation of non-axisymmetric solutions that bifurcate from the axisymmetric family at specific rotation rates. To validate our numerical procedure, we have reproduced these bifurcation points, which correspond to conditions of marginal stability (*λ*= 0) where the energy functional ceases to be a minimum relative to non-axisymmetric perturbations of a given mode number *n*. Results are shown in Fig. 4, where in the upper plot the bifurcation points for perturbations with mode numbers *n* = 2,3,4 and 5 are indicated on the curve of Ω_*_ versus L_*_. The numerical values agree with those given by Brown and Scriven for *n* = 2,3, and 4 to three decimal places; they were unable to compute the *n* = 5 bifurcation because of their use of spherical coordinates, which preclude the computation of highly-dimpled shapes that are non-convex relative to the origin. The results for *n* = 2 also agree with those given by Heine to five decimal places; Heine also describes a bifurcation to an *n* = 6 perturbation on the extension of the solution curves to self-intersecting drops for L_*_ > 1.1194. In the middle plot of Fig. 4 the values of *λ* are shown for the first five non-axisymmetric perturbations. Values of L_*_ for which *λ* is positive correspond to stable modes, while negative values of *λ* correspond to instabilities. The points where the curves cross the axis *λ*= 0 correspond to the bifurcation points indicated in the upper figure. For *n* = 2: Ω_*_ = 0.5599, L_*_ = 0.3016 ; for *n* = 3 : Ω_*_ = 0.7071, L_*_ = 0.4944 ; for *n* = 4 ; Ω_*_ = 0.7536, L_*_ = 0.7099 ; and for *n* = 5, Ω_*_ = 0.7239, L_*_ = 1.0235. The rotating drops are also unstable to a “decentering” perturbation with *n* = 1. The non-rotating spherical drop (Ω_*_ = 0) is marginally stable (*λ*= 0) to an arbitrary translation of the drop’s position, which for small translations corresponds to an *n* = 1 perturbation of the drop shape. This mode is destabilized (*λ*< 0) with finite rotation, where the effects of centripetal acceleration cause a slightly off-axis drop to drift outwards.

As discussed by Brown and Scriven, the stability of the driven drops (Ω_*_ = constant) and isolated drops (L_*_ = constant) to non-axisymmetric perturbations (*n* > 0) are identical, since the perturbed moment of inertia vanishes for non-axisymmetric perturbations. For the case of axisymmetric perturbations (*n* = 0), the stability results do differ, as shown in the lower plot in Fig. 4. The driven drop is unstable to an axisymmetric disturbance at the limit point [23] of the solution branch where Ω_*_ reaches its maximum value, with Ω_*_ = 0.7540, L_*_ = 0.7291. The isolated drop is stable with positive values of *λ* that, for each value of L_*_, are larger than those for the driven drop, as expected from Eq. (33). No limit point with respect to L_*_ occurs on the solution branch, so there is no analogous axisymmetric instability for the isolated drop.

### 5.3 Linear Stability of the Toroids

**Fig. 5:**
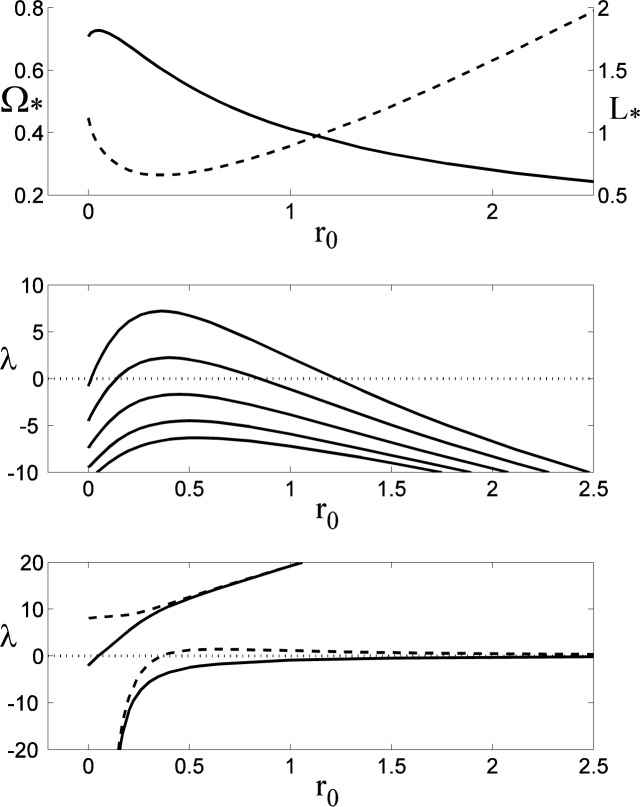
Linear stability of rotating toroidal drops. In the upper plot the rotation rate Ω_*_ (solid curve) and angular momentum L_*_ (dashed curve) for the base state are shown as functions of the inner radius *r*_0_. In the middle plot the least stable values of *λ* as a function of *r*_0_ are shown for *n* =1,2,3,4, and 5 (bottom to top). In the lower plot the two least stable values of *λ* for axisymmetric disturbances (*n* = 0) are shown for driven (solid curve) and isolated (dashed curve) toroidal drops.

The linear stability of the toroidal family of rotating drops is shown in Fig. 5. The upper plot gives the parametric relation between the rotation rate Ω_*_ (solid curve) and the angular momentum L_*_ (dashed curve) of the base state as functions of the dimensionless inner radius *r*_0_. As the inner radius tends to zero, the rotation rate decreases (over a short interval), and the angular momentum increases. The opposite is true as the inner radius becomes large, and there is a maximum value of Ω_*_ and a minimum value of L_*_ as *r*_0_ varies from zero to infinity. The middle plot shows the lowest eigenvalues for *n* = 1 to *n* = 5 for 0 < *r*_0_ < 2.5. For small values of *r*_0_ toroidal drops are unstable for all five mode numbers, but as *r*_0_ increases the stability of these modes increase and reach maxima near *r*_0_ = 0.5, where only the first three modes are unstable. With further increases of inner radius, the modes are all destabilized and the toroidal drop is unstable to higher and higher mode numbers; the trends indicated for the lowest five modes are also observed for higher mode numbers *n* and larger inner radii *r*_0_. The lower plot in Fig. 5 shows the lowest two eigenvalues for axisymmetric modes (*n* = 0) for the case of driven toroidal drops (solid curves) and isolated drops (dashed curves). As expected from the previous discussion of Eq. (33), the isolated drops are more stable than the driven drops in each case, although for large values of *r*_0_ the eigenvalues become nearly identical. For small values of *r*_0_ the difference is more pronounced. The lowest eigenvalues both become very large and negative as *r*_0_ tends to zero, but the lowest mode for the driven drop remains slightly unstable for large *r*_0_, whereas the isolated drop becomes stable near *r*_0_ = 0.5 and then deceases in magnitude for large *r*_0_. The second lowest modes are both stabilized with increasing *r*_0_, although the driven drop is initially unstable for small *r*_0_. There are two axisymmetric, neutrally-stable modes (*λ*= 0). For the driven drop the neutral mode corresponds to a limit point on the solution branch in which L_*_ is regarded as a function of Ω_*_. For the isolated drop the neutral mode corresponds to a limit point on the solution branch in which Ω^*^ is regarded as a function of L_*_. In the top plot in Fig. 5 these points corresponds to extremal values of Ω_*_ and L_*_ regarded as functions of *r*_0_. These results indicate that the family of rotating driven toroidal drops is entirely unstable, both to axisymmetric and non-axisymmetric disturbances, with an increasing number of non-axisymmetric instabilities with increasing *r*_0_. The same is true for non-axisymmetric disturbances to the isolated drop, although in that case axisymmetric perturbations are stable for large enough values of *r*_0_.

**Fig. 6:**
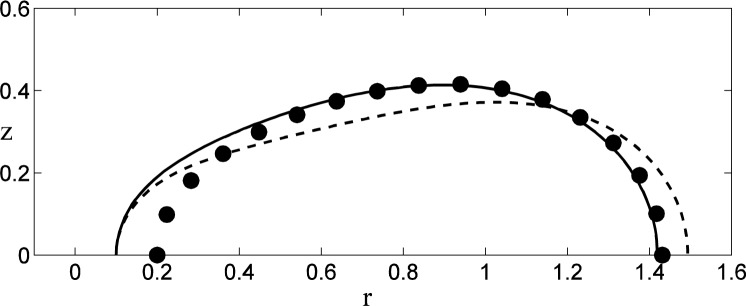
Geometry of the axisymmetric perturbations to the base state (solid dots) for *r*_0_ = 0.2. The lowest mode (solid curve) and the second lowest mode (dashed curve) are both normalized to give similar displacements at the inner radius, and the size of the perturbations has been exaggerated for visual purposes.

The geometry of the unstable axisymmetric modes is shown in Fig. 6 for a driven drop with *r*_0_ = 0.2, where the perturbed shapes for the lowest two modes are shown superimposed upon the base state (solid dots). The lowest mode (solid curve) represents a distortion of the shape that occurs predominantly at small radii, leaving the outer portion of the drop unaffected. Loosely speaking, this mode represents an instability driven by a change in the major radius of the torus. The second lowest mode (dashed curve) represents a perturbation that changes the ellipticity of the cross-section, with distortions at the inner and outer radii that are accompanied by a distortion of opposite sign at intermediate radii that preserves the net volume.

#### 5.3.1 Rayleigh Instability Analogy

**Fig. 7:**
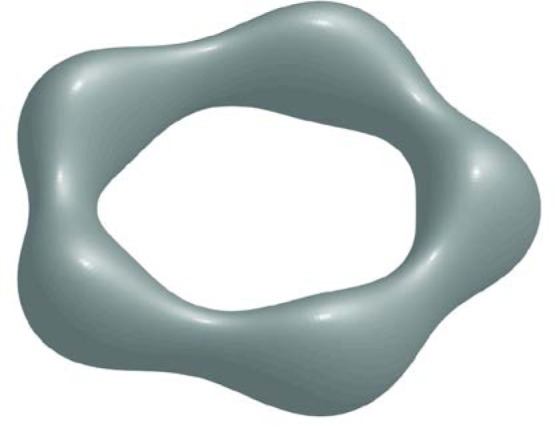
Example of a non-axisymmetric (*n* = 5) neutral (*λ* = 0) eigenmode. Here Ω_*_ = 0.37 and L_*_ = 1.03; the eigenmode is shown superimposed on the axisymmetric base state with a large amplitude for visual purposes. The shape is reminiscent of the evolving toroidal shapes observed by McGraw et al. shown in Figs. 3 and 4 of Ref. [12].

With increasing values of angular momentum L_*_ drops are subject to an increasing number of non-axisymmetric instabilities; only the most dangerous modes in the first five families (*n* =1,2,3,4,5) of instabilities are shown in the middle plot of Fig. 5. An example of a non-axisymmetric instability is shown in Fig. 7, corresponding to the neutral instability for *n* = 5 that occurs near *r*_0_ = 1.2 in Fig. 5.

For toroidal drops with a large major radius, an interpretation of these high-wavenumber modes is possible in terms of a classical surface-tension-driven instability. For example for large values of L_*_ the cross-section of the drops become more and more circular, and the drops increasingly resemble a circular torus. For large values of the effective major radius of the drop, the non-axisymmetric instabilities are then analogous to the capillary-driven Rayleigh instabilities [24] of an equivalent cylinder of length *2πR_M_* and radius *r_m_*, where *R_M_* = (*r*_1_ + *r*_0_)/2 and *r_m_* = (*r*_1_ − *r*_0_)/2 ≪ *R_M_* are the major and minor radii of the torus based on the inner and outer radii *r*_0_ and *r*_1_. The onset of the Rayleigh instability occurs for a perturbation whose wavelength *λ_R_* is equal to the circumference *2πr_m_* of the cylinder [24]. For an effective cylinder length *L_c_* = 2*πr_m_* we therefore anticipate neutral modes with mode number *n_R_* such that *n_R_λ_R_* = *L_c_*, or *n_R_* = *R_M_/r_m_.* We can readily compute values for *R_M_* and *r_m_* from the numerical solution in this regime and compare this estimate for *n_R_* with the numerically-computed values of *n* that have crossings at *λ_n_* = 0. For example, in Fig. 5 the *n* = 4 mode with L_*_ = 0.814 is neutrally stable (*λ*_4_ = 0). For this drop, the computed radii are *R_M_* = 1.2706 and *r_m_* = 0.4206, which gives the estimate *n_R_* = 3.02. The estimate becomes more accurate for drops with larger values of *R_M_*; for a drop with L_*_ = 1.921 we find *R_M_* = 2.7159 and *r_m_* = 0.2803, giving *n_R_* = 9.69. The corresponding numerical results show that the perturbation with *n* = 10 is neutrally stable under these conditions. Some numerical results are summarized in Table 1. The analytical approximation may also be obtained directly from the Jacobi equation (30) in this regime: the dominant balance for *λ* = 0 is found to be

$$\frac{\gamma n^{2}}{R^{2}}W\approx \gamma \psi_{s}^{2}W,\;W_{s}(0)=W_{s}(S_{T})=0,$$ (36)

where for the toroidal base state we have *R*(*s*) ≈ *R_M_*, *S_T_* ≈ *πr_m_* and *ψ_s_* ≈ 1/*r_m_.* The resulting eigenmode *W*(*s*) is approximately constant, with $n^{2}=R_{M}^{2}/r_{m}^{2}$ as in Rayleigh’s analysis.

### 5.4 Driven Drops for Δρ *<*0

For a rotating spheroidal drop inside a denser medium (Δρ *<* 0) the effective centrifugal force at the equator is inward, and the drops are elongated at the poles rather than the equator; we designate the resulting shapes as prolate spheroids. A dimensionless rotation rate Ω*_P_* for the prolate solutions is then defined as

$$\Omega_{P}^{2}=\frac{-\Delta \rho \Omega^{2}R_{0}^{3}}{8\gamma }.$$ (37)

We consider only the case of driven drops. An analytic solution in this case was derived by Rosenthal [33] and Princen [34] in terms of incomplete elliptic integrals and is summarized in § 7.1. We have also implemented the previously-described numerical procedure for the base state in this case as well in order to facilitate the stability calculations.

**Fig. 8:**
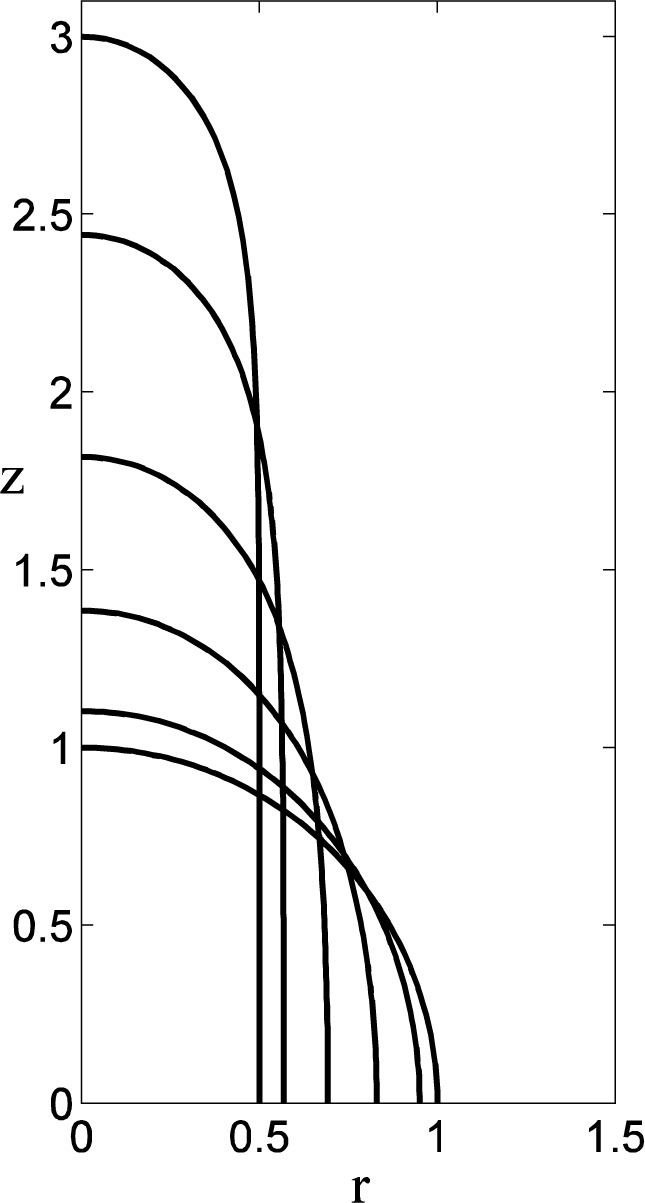
Prolate equilibrium shapes (Δ*ρ<*0) for various rotation rates Ω*_P_.* For increasing polar radii *z_P_*, the curves correspond to *z_P_* =1 (the spherical case with equatorial radius *r_E_*=1 and Ω*_P_*= L_*_ =0),* z_P_*=1.1029 (*r_E_* = 0.9502, Ω,*_P_*=0.4, L_*_ = 0.1516), *z_P_* =1.3854 (*r_E_* = 0.8299, Ω*_P_* = 0.8, L_*_ = 0.2361), *z_P_* =1.8176 (*r_E_* = 0.6920, Ω*_P_* = 1.2, L_*_ = 0.2591), *z_P_* = 2.4423 (*r_E_* = 0.5680, Ω*_P_* = 1.6, L_*_ = 0.2557), *z_P_* = 3 (*r_E_* = 0.5, Ω*_P_* = 2, L_*_ = 0.2461).

In Fig. 8 we show the evolution of the prolate spheroidal shapes as the rotation rate Ω*_P_* is increased. For Ω*_P_* = 0 the equilibrium is a spherical drop, and with increasing Ω*_P_* the equilibria tend to approximately cylindrical shapes that are terminated by roughly spherical caps. The equatorial radius *r_E_* decreases monotonically and the polar radius *z_P_* increases monotonically with increasing rotation rate, consistent with the imposed constraint of equal volumes for the family. Some numerical results are given in Table 2. For large rotation rates approximate expressions for the equatorial radius $\left(r_{E}^{A}\right)$ and the polar radius $\left(z_{\text{tip}}^{A}\right)$ can be obtained from an asymptotic evaluation of the elliptic integrals (see § 7.1); the corresponding results are also given in Table 2.

**Fig. 9:**
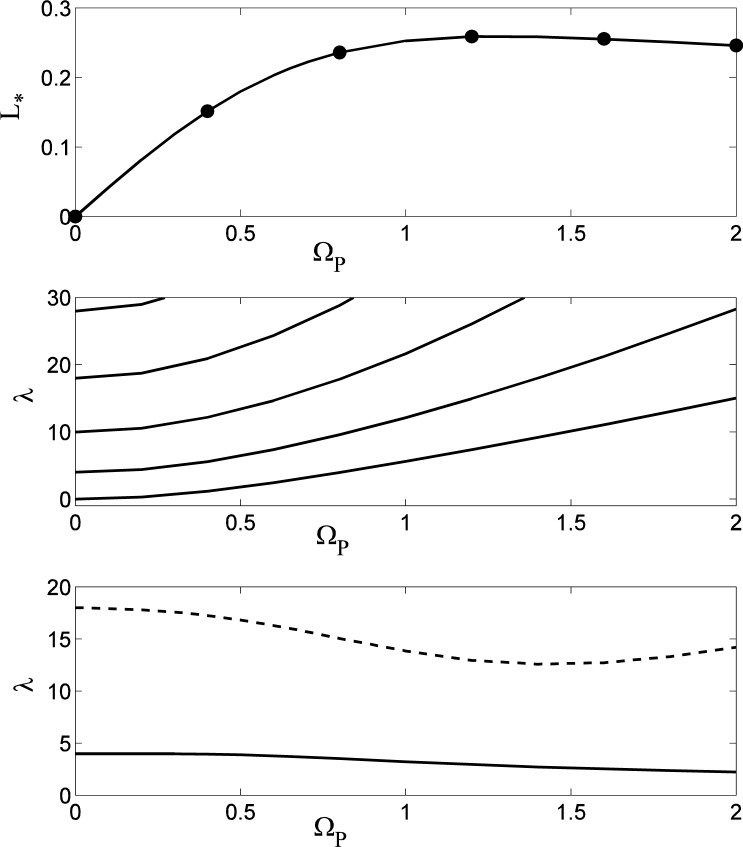
Linear stability of rotating prolate drops. In the upper plot the angular momentum L_*_ of the base state is given as a function of the rotation rate Ω*_P_*; solid dots correspond to the shapes given in Fig. 8. In the middle plot the least stable values of *λ* as a function of Ω*_P_* are shown for non-axisymmetric perturbations with *n* = 1,2,3,4, and 5 (bottom to top). In the lower plot the two least stable values of *λ* for axisymmetric disturbances (*n* = 0) are shown.

Numerical calculations for the linear stability of the prolate drops are shown in Fig. 9. For both axisymmetric perturbations (lower plot) and non-axisymmetric perturbations (middle plot) the drops are found to be stable (*λ* > 0). The stationary drop with Ω*_P_* = 0 is again neutrally stable (*λ* = 0) to an *n* = 1 mode that represents a lateral translation of the drop. For very large rotation rates the most dangerous axisymmetric mode is becoming decreasingly stable; the other modes are apparently increasingly stable for increasing rotation rates.

As the rotation rate increases the drops become quite elongated with cylindrical mid-sections; it is therefore interesting to consider the possibility of a Rayleigh instability to axisymmetric perturbations with suitable wavelengths. We note that rotation about the cylindrical axis is known to stabilize the Rayleigh instability of an infinite cylinder if Δ*ρ <* 0. For example, Gillis and Kaufman [25] show that the rotating cylinder is stable if

$$R_{C}^{2}k^{2}+n^{2}-1\geq \frac{\Delta \rho \Omega^{2}R_{C}^{3}}{\gamma },$$ (38)

where *R_C_* is the cylinder radius, and *k* and *n* are the axial and azimuthal wavenumbers. Axisymmetric modes (*n* = 0) are stable for disturbances with $R_{C}^{2}k^{2}\geq 1+\Delta \rho \Omega^{2}R_{C}^{3}/\gamma$, so that for Δ*ρ >* 0 the range of stable wavenumbers *k* decreases with increasing rate of rotation, and for Δ*ρ <* 0 this range increases with increasing rate of rotation. If $\Delta \rho \Omega^{2}R_{C}^{3}/\gamma <-1$ all wavenumbers are stable to axisymmetric modes. Non-axisymmetric modes with *n* ≥ 1 are also stable for all wavenumbers if Δ*ρ <* 0.

The result (38) can also be obtained directly from the Sturm-Liouville equation (30) by setting *ψ* = −*π*/2, *s* = *z*, *R*= *R_C_*, and *μ* = 0, giving

$$-\left\{1+\frac{\Delta \rho \Omega^{2}R_{C}^{3}}{\gamma }\right\}W_{1}+(k^{2}R_{C}^{2}+n^{2})W_{1}=\left(\frac{\gamma \lambda }{R_{C}^{2}}\right)W_{1},$$ (39)

where we have expressed the eigenmode as *W*^(1)^(*s,θ*) = *W*_1_ exp(*ikz + inθ*). The volume constraint (29) with *S_T_* = 2*π*/*k* is identically satisfied for *W*^(1)^ (*s, θ*) of this form. The stability condition in Eq. (38) is thus equivalent to our stability condition *λ* ***>*** 0.

**Fig. 10:**
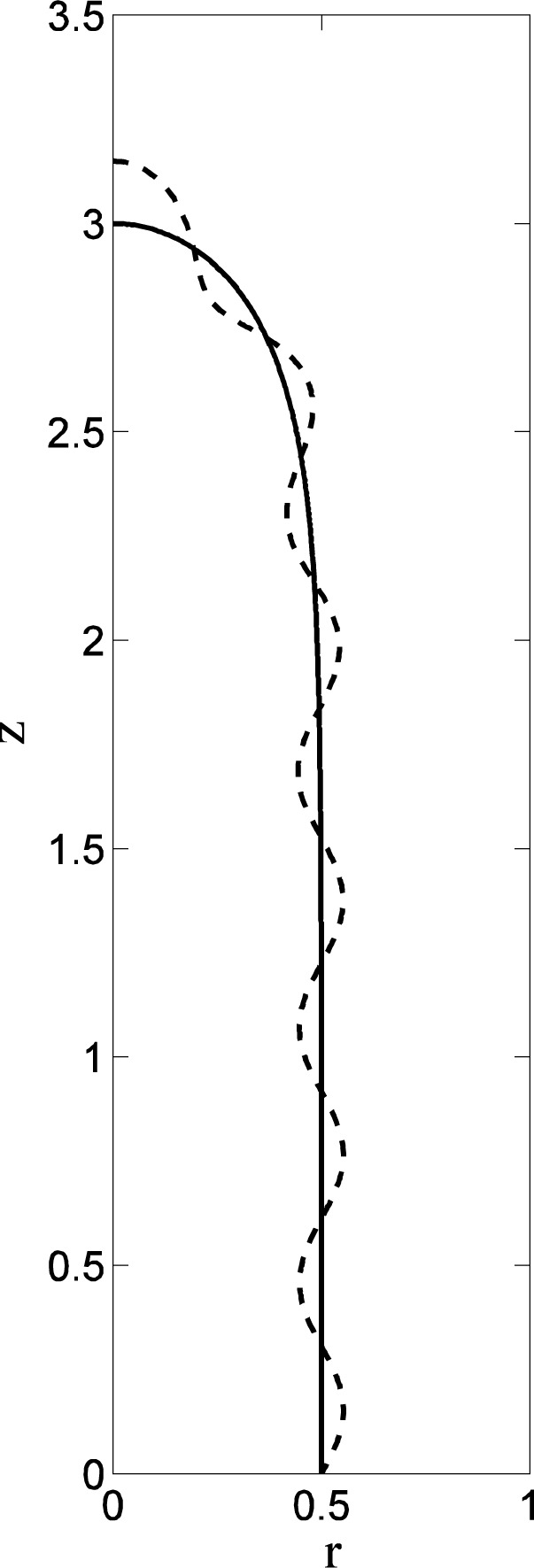
Prolate spheroidal solution for Ω*_P_* = 2. The upper half of a cross-section of the base state is shown as the solid curve, and a high-order stable eigenmode is shown as the dashed curve. The amplitude of the eigenmode has been exaggerated for visual purposes.

An example is shown in Fig. 10, where a (quite stable) higher-order axisymmetric eigenmode is shown superimposed on the prolate solution with Ω*_p_* =2. The equatorial radius is *r_E_* ≈ 0.5*R*_0_, and the prolate solution is elongated enough that near its midsection (*r* ≈ *r_E_*) the eigenmode is approximately sinusoidal with a computed wavelength of 2*π*/*k* = 0.6124*R*_0_. If we take *R_C_* = *r_E_* the cylindrical relation (39) then gives the result $\gamma \lambda /R_{0}^{2}=117.40$, which compares well with the computed result $\gamma \lambda /R_{0}^{2}=117.47$ that is obtained for the prolate spheroid withΩ*_p_* =2.

## 6. Discussion

We have computed solutions for axisymmetric equilibrium shapes of spheroidal and toroidal drops or bubbles that correspond to extrema of an energy functional containing surface energy and rotational energy contributions, subject to a volume constraint. Examination of the second variation of the energy functional then determines whether the drops are stable, representing energy minima, or instead represent unstable saddle points or energy maxima. An alternate approach is to determine the linear stability of equilibrium shapes by solving the hydrodynamic equations of motion as given by Newton’s law, which provides a dynamical growth rate for normal mode solutions. For example, Pairam and Fernández-Nieves ([11], see also [26]) are able to interpret their experimental observations of the breakup of toroidal drops by comparing with the theoretical results of Tomotika [27] for the fastest-growing instability of a cylindrical thread of viscous liquid surrounded by another viscous fluid. A number of other authors have discussed the dynamic instability of toroidal drops based on approximate base states that are assumed to have circular cross sections [28, 29], or have observed or simulated the temporal evolution of arbitrary (non-equilibrium) toroidal shapes [13, 26, 30, 31]. Our approach focuses on the accurate computation of bifurcation points for self-consistent equilibrium shapes. As discussed by Brown and Scriven [9], the role of viscosity in determining the linear stability of rotating drops by solving the hydrodynamic governing equations can lead to subtle distinctions between “ordinary stability” and “secular instability,” wherein an equilibrium that is stable according to the inviscid equations of motion is destabilized by the inclusion of viscous effects [6, 32]. The stability results that we compute based on energy minimization correspond to the viscous case in this context.

## Figures and Tables

**Table 1:** Large *n* neutral modes for the toroidal solutions.

Ω_*_	L_*_	*r*_0_	*r*_1_	*n_R_*	*n*(*λ_n_* = 0)
0.44442	0.81402	0.85006	1.69117	4	3.02
0.37100	1.02696	1.22376	1.96368	5	4.31
0.30048	1.40510	1.77469	2.41500	7	6.54
0.24672	1.92093	2.43561	2.99616	10	9.69

**Table 2:** Base state parameters for the prolate spheroidal solutions.

Ω*_P_*	L_*_	*r_E_*	*z*_tip_	$r_{E}^{A}$	$z_{\text{tip}}^{A}$
0.00000	0.00000	1.00000	1.00000	–	–
0.80000	0.236121	0.829926	1.38539	0.857845	1.39306
1.28510	0.259531	0.665097	1.92606	0.665299	1.92607
2.00000	0.246142	0.499979	3.00000	0.499979	3.00000
2.40000	0.236130	0.442774	3.69569	0.442774	3.69569

## 7. Appendix

### 7.1 Elliptic Integral Formulation for Prolate Spheroids

For spheroidal solutions, the explicit first integral of the Euler equation (8) has a vanishing constant of integration. The oblate spheroidal solution, for Δ*ρ* > 0, was obtained in terms of elliptic integrals by Chandrasekhar [7], and for the prolate spheroidal solution, for Δ*ρ* < 0, by Rosenthal [33] and Princen [34]. Here we summarize the solution for the prolate spheroid to obtain the asymptotic solution used in Table 2.

Evaluating Eq. (8) at *r* = *r*_1_ where *ψ* = −*π*/2 so that *f*_r_ → −∞ gives

$$1=\frac{Pr_{1}}{2\gamma }+\sum ,\;\sum =\frac{\Delta \rho \Omega^{2}r_{1}^{3}}{8\gamma },$$ (A1)

where Σ is a dimensionless rotation rate used by Chandrasekhar [7], who then goes on to obtain solutions for Σ > 0 which correspond to the oblate spheroid. Here we consider the range −1/2 < Σ < 0, corresponding to solutions for the prolate spheroid. The dimensionless rotation rates Σ and Ω*_P_* are related by $\Sigma =-{\left(r_{1}/R_{0}\right)}^{3}\Omega_{P}^{2}$.

Scaling by the equatorial radius, with $\bar{r}=r/r_{1}$, the first integral can now be expressed as

$$\frac{f_{\bar{r}}}{\sqrt{1+f_{\bar{r}}^{2}}}=(1-\sum )\bar{r}+\Sigma {\bar{r}}^{3},$$ (A2)

which can be solved for $f_{\bar{r}}$ and integrated to give

$$f(\bar{r})=\int_{\bar{r}}^{1}\frac{y(1-\Sigma +\Sigma y^{2})}{{\left[1-y^{2}{\left(1-\Sigma +\Sigma y^{2}\right)}^{2}\right]}^{1/2}}dy.$$ (A3)

Another change of variables to *x* = *y*^2^, with *dx* = 2*ydy*, gives

$$f(\bar{r})=\frac{1}{2}\int_{{\bar{r}}^{2}}^{1}\frac{(1-\Sigma +\Sigma x)dx}{\sqrt{\left[1-x{\left(1-\Sigma +\Sigma x\right)}^{2}\right]}}.$$ (A4)

In the integral above, the argument of the radical can be simplified as Σ^2^(*c* − *x*)(*b* − *x*)(*a* − *x*) where

$$a=\frac{(\Sigma -2)-{\left[\Sigma (\Sigma -4)\right]}^{1/2}}{2\Sigma },\;b=\frac{(\Sigma -2)+{\left[\Sigma (\Sigma -4)\right]}^{1/2}}{2\Sigma },\;c=1.$$ (A5)

For −1/2 < Σ < 0, the roots are all real with 1 < *b* < *a*.

Therefore, Eq. (A4) can be rewritten as

$$f(\bar{r})=\frac{1}{2|\Sigma |}\int_{{\bar{r}}^{2}}^{1}\frac{(1-\Sigma +\Sigma x)dx}{{\left[(1-x)(b-x)(a-x)\right]}^{1/2}}=\frac{\left(1-\Sigma \right)}{2|\Sigma |}S_{0}-\frac{1}{2}S_{1}.$$ (A6)

where

$$S_{0}=\int_{{\bar{r}}^{2}}^{1}\frac{dx}{{\left[(1-x)(b-x)(a-x)\right]}^{1/2}},$$ (A7)

$$S_{1}=\int_{{\bar{r}}^{2}}^{1}\frac{xdx}{{\left[(1-x)(b-x)(a-x)\right]}^{1/2}}.$$ (A8)

The volume of the prolate spheroid also has an analytical expression given by

$$V=\pi (1-\Sigma )J_{1}+\pi \Sigma J_{2},$$ (A9)

where

$$J_{1}=-\frac{S_{1}}{\Sigma },\;J_{2}=\frac{2}{3\Sigma^{2}}+{\left(1-\Sigma \right)}^{2}\frac{S_{0}}{3\Sigma^{3}}+\frac{4(1-\Sigma )S_{1}}{3\Sigma^{2}}.$$ (A10)

From Gradshteyn and Ryzhik [35], with $a>b>1>{\bar{r}}^{2}$, the integrals *S*_0_ and *S*_1_ can be expressed in terms of incomplete elliptic integrals,

$$S_{0}=\frac{2}{\sqrt{a-1}}F(\phi ,k),$$ (A11)

$$S_{1}=\frac{2}{\sqrt{a-1}}\left\{F(\phi ,k)+(a-1)E(\phi ,k)\right\}-2{\left[\frac{\left(a-{\bar{r}}^{2})(1-{\bar{r}}^{2}\right)}{\left(b-{\bar{r}}^{2}\right)}\right]}^{1/2}.$$ (A12)

Here *F*(*ϕ*, *k*) and *E*(*ϕ*, *k*) are the incomplete elliptic integrals of the first and second kind respectively given by

$$F(\phi ,k)=\int_{0}^{\phi }\frac{d\theta }{\sqrt{1-k^{2}\sin^{2}}\theta },\;E(\phi ,k)=\int_{0}^{\phi }\sqrt{1-k^{2}\sin^{2}\theta }d\theta$$ (A13)

where

$$\sin^{2}\phi =\frac{1-{\bar{r}}^{2}}{b-{\bar{r}}^{2}},\;k^{2}=\frac{a-b}{a-1}.$$ (A14)

The parameter *k* is the elliptic modulus or eccentricity that must satisfy 0 < *k*^2^ < 1 and *ϕ* is called the argument of the normal elliptic integral and is usually taken to be 0 < *ϕ* < *π*/2.

Therefore by inverting Eq. (A14) and recalling that $\bar{z}=f(\bar{r})$, we have the parametric representation

$$\bar{r}(\phi )=\sqrt{\frac{1-b\;\sin^{2}\phi }{1-\sin^{2}\phi }},$$ (A15)

and

$$\bar{z}(\phi )=\frac{\left(1-\Sigma \right)}{2|\Sigma |}S_{0}\;(\phi )-\frac{1}{2}S_{1}\;(\phi ),$$ (A16)

for 0 ≤ *ϕ* ≤ *ϕ_M_*, with sin ^2^*ϕ_M_* = 1/*b*. A numerical solution of these parametric representations was used to validate the numerical base state calculations shown in Fig. 8.

Approximate results for the tip position (*r* = 0, z = z_t_*_ip_*) can be obtained in the high rotation limit, which corresponds to Σ → −1/2. In this limit *b* → 1, *k*^2^ → 1, and *ϕ* = *ϕ_M_* → *π*/2, and asymptotic expansions of the incomplete elliptic integrals are possible [36]. For Σ = −1/2 + *ε* we have used the approximations

$$F(\phi_{M}(\varepsilon ),k(\varepsilon ))\approx \frac{1}{2}\log\left(\frac{18}{\varepsilon }\right)-\ln(2+\sqrt{3}),\;E(\phi ,k)\approx 1.$$ (A17)

Using these expressions in Eq. (A11) and Eq. (A12) to evaluate Eq. (A15) and Eq. (A16) gives the approxixmate results shown in Table 2.

### 7.2 Matrix Solution

Discretization of the Sturm-Liouville equations for the driven drop using centered differences on a uniform grid with *N* mesh points produces a standard matrix eigenproblem that can be solved with conventional techniques for the non-axisymmetric case with *n* > 0, since the volume constraint is automatically satisfied. The corresponding problem for the axisymmetric case with *n* =0 is more complicated since the volume constraint needs to be satisfied explicitly, which couples the eigenvalue equations with a linear equation that results from applying a quadrature formula to Eq. (29). We sketch an approach to this problem based on work by Golub [37].

We write the approximation to an eigenmode as **w***^T^* = (*w*_1_,…, *w_N_*), where “*T*” denotes the matrix transpose. We define a discrete inner product

$$<u,v>=\frac{R_{1}u_{1}v_{1}}{2}+R_{2}u_{2}v_{2}+\ldots R_{N-1}u_{N-1}v_{N-1}+\frac{R_{N}u_{N}v_{N}}{2}$$ (A18)

based on the trapezoidal quadrature rule for an integrand *R*(*s*)*u*(*s*)*v*(*s*). The constraint in Eq. (29) is then approximated by <**1**,**w**>=0, where **1** is the constant vector with **1***^T^* = (1,…,1). The discrete approximation to the second variation can be written in the form $E^{\left(2\right)}=<w,ℍw>$, where ℍ is the finite difference approximation to the Sturm-Liouville operator on the left hand side of Eq. (30); ℍ is a tridiagonal matrix with <u,ℍv>=<ℍu,v>. If we let **h***^T^* = (*R*_1_/2, *R*_2_,…, *R_N_*_−1_, *R_N_*/2), then we may also write <**1**,**w**> = **h***^T^***w** as a conventional matrix inner product. The constraint equation is then treated by introducing an explicit projection onto the subspace <**1**,**w**> = 0, defined by the matrix

$$\mathbb{P}=I-\frac{1h^{T}}{h^{T}1},$$ (A19)

where I is the identity matrix and the outer product **1h***^T^* is a rank-one matrix. ℙ satisfies ℙ1=0 and ℙw=0 if <**1**,**w**>=0, and is symmetric with respect to the inner product: <u,ℙv>=<ℙu,v>, with ${\mathbb{P}}^{2}=\mathbb{P}$.

The diagonalization of *E*^(2)^ on the subspace <**1**,**w**> = 0 is achieved by computing the *N* normal modes **w***_j_* that satisfy the conventional symmetric eigenvalue problem ${\mathbb{P}}^{T}ℍ\mathbb{P}w_{j}=\lambda_{j}w_{j}$. We note that since ℙ1=0, the vector **1** is an eigenmode corresponding to *λ* = 0. The remaining modes are orthogonal to **1** and so satisfy the constraint < **1**,**w***_j_*> =0, with $\mathbb{P}w_{j}=w_{j}$. For these modes $E^{\left(2\right)}=[w_{j}]=<w_{j},ℍw_{j}>=<\mathbb{P}w_{j},ℍ\mathbb{P}w_{j}>=<w_{j},{\mathbb{P}}^{T}ℍ\mathbb{P}w_{j}>=\lambda_{j}<w_{j},w_{j}>$, which diagonalizes the discrete second variation, *E*^(2)^[∑*a_j_* **w***_j_*] = ∑*λ_j_* |*a_j_*|^2^ <**w***_j_*, **w***_j_*>. We note that by using the explicit expression (A19) for ℙ, the eigenvalue problem ${\mathbb{P}}^{T}ℍ\mathbb{P}w_{j}=\lambda_{j}w_{j}$ can be re-written as

$$ℍw_{j}=\lambda_{j}w_{j}+\left[\frac{h^{T}ℍw_{j}}{h^{T}1}\right]1,$$ (A20)

if <**1**,**w**> = 0, which is a discrete version of Eq. (30) with $\left(h^{T}ℍw_{j})/(h^{T}1\right)$ providing a discrete approximation to the Lagrange multiplier *µ_j_*.

For the non-axisymmetric isolated drop, the discretization of Eq. (34) using centered differences for the differential operator and a quadrature formula for the integral operator produces a dense matrix rather than a tridiagonal matrix, but this problem is still amenable to solution with a conventional eigensolver. For the axisymmetric problem the volume constraint is treated by subspace projection in the same manner as for the driven drop, and the resulting diagonalization again allows the stability of the drop to be determined from the signs of the eigenvalues of the corresponding normal modes.

### 7.3 ODE Solution

A shooting procedure can be used to compute numerical solutions for the unperturbed drop, and to solve the associated stability problem. For a given value of Ω, the base state can be computed by solving the system of ODE’s

$$R_{s}=\cos\psi ,$$ (A21)

$$Z_{s}=\sin\psi ,$$ (A22)

$$\gamma \psi_{s}=\frac{-\gamma \sin\psi }{R}-P-\frac{\Delta \rho \Omega^{2}}{2}R^{2},$$ (A23)

$$V_{s}=4\pi R(s)Z(s)\cos\psi ,$$ (A24)

$$Y_{s}-4\pi \Delta \rho {\left[R(s)\right]}^{3}Z(s)\cos\psi .$$ (A25)

to determine the drop shape *r* = *R*(*s*) and *z* = *Z*(*s*) for 0 <*s* <*S_T_*, and the Lagrange multiplier *P* for the volume constraint. Here *V*(*s*) and *Y*(*s*) are introduced in order to facilitate computation of the drop’s volume *V*(*S_T_*) and moment of inertia *I = Y*(*S_T_*).

For the shooting procedure in the spheroidal case appropriate initial conditions at *s* = 0 are *R*(0) = 0, *Z*(0) = *Z*_0_, *ψ*(0) = 0, *V*(0) = 0 and *Y*(0) = 0 where *Z*_0_, is the (unknown) polar radius at *r* = 0. At the equator *R*(*S_T_*) = *r*_1_, *Z*(*S_T_*) = 0, and *ψ*(*S_T_*) = −π/2. The shooting procedure uses provisional values of *Z*_0_, and *P*, and integrates from *s* = 0 until a value *s* = *S_T_* where *Z*(*S_T_*) = 0. The desired conditions *ψ*(*S_T_*) = −π/2 and *V*(*S_T_*) = *V*_0_ will generally not be satisfied with this choice of *Z*_0_ and *P*, so these values are updated by a root solver that drives *ψ*(*S_T_*) and *V*(*S_T_*) to their required values. Upon convergence we also then have values for the equatorial radius *R*(*S_T_*) = *r*_1_, the moment of inertia $ℐ=Y(S_{T})$, and the angular momentum L=ℐΩ.

For the toroidal case we have *R*(0) = *r*_0_ and *R*(*S_T_*) = *r*_1_, with *Z*(0) = *Z*(*S_T_*) = 0, and *ψ*(0) = *π*/2 = −*ψ*(*S_T_*). In this case we instead start with provisional values for *R*(0) and *P*, and iterate on these values until *ψ*(*S_T_*) = −*π*/2 and *V*(*S_T_*) = *V*_0_.

To solve the stability problem for the non-axisymmetric driven drop we append the equation

$$\gamma w_{ss}=-\gamma \frac{\cos\psi }{R}w_{s}+\left\{-\gamma \psi_{s}^{2}-\gamma \frac{\sin^{2}\psi }{R^{2}}+\Delta \rho \Omega^{2}R^{2}\frac{\sin\psi }{R}\right\}w-\gamma \frac{n^{2}}{R^{2}}w-\lambda w,$$ (A26)

with initial conditions *w*(0) = 1, *w_s_*(0) = 0. Provisional values of *λ* are used to drive *w_s_*(*S_T_*) to zero, so that the perturbation has the correct Neumann boundary conditions. Initial guesses for *λ* are obtained from the matrix solution.

For the axisymmetric drop we append the equations

$$\gamma w_{ss}=-\gamma \frac{\cos\psi }{R}w_{s}+\left\{-\gamma w_{s}^{2}-\gamma \frac{\sin^{2}\psi }{R^{2}}+\Delta \rho \Omega^{2}R^{2}\frac{\sin\psi }{R}\right\}w-\gamma \frac{n^{2}}{R^{2}}w-\lambda w-\mu ,$$ (A27)

$$V_{s}^{\left(1\right)}=4\pi R(s)w(s),$$ (A28)

with initial conditions *w*(0) = 1, *w_s_*(0) = 0, and *V*^(1)^(0) = 0. Provisional values of *λ* and *µ* are used to drive *w_s_*(*S_T_*) and *V*^(1)^(*S_T_*) to zero, so that the perturbation has the correct Neumann boundary conditions and satisfies the volume constraint. Initial guesses for *λ* and *µ* are obtained from the matrix solution. For the spheroidal case, to avoid the singularity at *s* = 0 in Eq. (A27) we compute a series solution for small *s* (see Appendix 7.4), and start the numerical integration at a small positive value of *s* using values from the series solution.

For the isolated non-axisymmetric drop, the integro-differential equation (34) is converted to an ODE by replacing the integral term by an auxiliary variable during the shooting procedure. We set

$$B=4\pi {\left(\Delta \rho \right)}^{2}\left(\frac{L^{2}}{{ℐ}^{3}}\right)\int_{0}^{S_{T}}{\left[R(s)\right]}^{3}w(s)d\theta ds$$ (A29)

and append ODE’s of the form

$$\gamma w_{ss}=-\gamma \frac{\cos\psi }{R}w_{s}+\left\{-\gamma \psi_{s}^{2}-\gamma \frac{\sin^{2}\psi }{R^{2}}+\Delta \rho \Omega^{2}R^{2}\frac{\sin\psi }{R}\right\}w+B{\left[R(s)\right]}^{2}-\gamma \frac{n^{2}}{R^{2}}w-\lambda w,$$ (A30)

$$Y_{s}^{\left(1\right)}=4\pi {\left(\Delta \rho \right)}^{2}\left(\frac{L^{2}}{{ℐ}^{3}}\right)R^{3}w.$$ (A31)

With the initial conditions *w*(0) = 1, *w_s_*(0) = 0, and *Y*^(1)^(0) = 0, we solve the ODEs and then iterate on *λ* and *B* to obtain *w_s_*(*S_T_*) = 0 and *Y*^(1)^(*S_T_*) = *B*.

Similarly, for the axisymmetric isolated drop we solve

$$\gamma w_{ss}=-\gamma \frac{\cos\psi }{R}w_{s}+\left\{-\gamma \psi_{s}^{2}-\gamma \frac{\sin^{2}\psi }{R^{2}}+\Delta \rho \Omega^{2}R^{2}\frac{\sin\psi }{R}\right\}w+B{\left[R(s)\right]}^{2}-\lambda w-\mu ,$$ (A32)

$$V_{s}^{\left(1\right)}=4\pi Rw,$$ (A33)

$$Y_{s}^{\left(1\right)}=4\pi {\left(\Delta \rho \right)}^{2}\left(\frac{L^{2}}{{ℐ}^{3}}\right)R^{3}w.$$ (A34)

With the initial conditions *w*(0) = 1, *w_s_*(0) = 0, *V*^(1)^(0) = 0, and *Y*^(1)^(0) = 0, we iterate on *λ*, *µ*, and *B* to obtain *w_s_*(*S_T_*) = 0, *V*^(1)^*(S_T_*) = 0, and *Y*^(1)^(*ST*) = *B.*

### 7.4 Taylor Series Expansions

For the spheroidal solutions, there is a singularity in the appended Sturm-Liouville equation Eq. (A27) where the arclength *s* = 0 at *r*_0_ = 0. The singular terms are avoided by employing a Taylor series expansions for the base state *R*(*s*), *Z*(*s*) and *ψ*(*s*) about *s* = 0, given by

$$R(s)=s-\frac{P^{2}}{24}s^{3}+\left[\frac{P^{4}}{1920}-\frac{P\Omega^{2}}{10}\right]s^{5}+\left[-\frac{P^{6}}{322560}+\frac{11P^{3}\Omega^{2}}{1680}-\frac{\Omega^{4}}{14}\right]s^{7}+O(s^{9}),$$ (A35)

$$Z(s)=Z_{0}-\frac{P}{4}s^{2}+\left[\frac{P^{3}}{192}-\frac{\Omega^{2}}{4}\right]s^{4}+\left[\frac{7P^{2}\Omega^{2}}{240}-\frac{P^{5}}{23040}\right]s^{6}+O(s^{8}),$$ (A36)

$$\psi (s)=-\frac{P}{2}s-\Omega^{2}s^{3}+\frac{P^{2}\Omega^{2}}{20}s^{5}+\left[-\frac{P^{4}\Omega^{2}}{840}+\frac{3P\Omega^{4}}{35}\right]s^{7}+O(s^{9}),$$ (A37)

which were found by solving the differential equations (A21)–(A23) with the appropriate initial conditions at *s* = 0.

The perturbation is expanded as

$$W(s)=w_{0}s^{\alpha }(1+w_{2}s^{2}+w_{4}s^{4}+\ldots ),$$ (A38)

where the coefficients of the odd powers of *s* are found to be zero due to symmetry. The remaining coefficients are determined by the method of Frobenius as follows.

Firstly, the non-axisymmetric drop, *n* ≥ 1 is considered. Here, the Sturm-Liouville equation Eq. (A26) at leading order in *s* gives an indicial equation for *α* with solution *α* = ±*n*. For regularity of solution, *α* = *n* is chosen. Solving for additional terms in the perturbation series at $O(s^{n})$ gives

$$w_{2}=\frac{1}{48(n+1)}\left[P^{2}(n+3)(n-2)-12\lambda \right],$$ (A39)

$$w_{4}=\frac{1}{23040(n+1)(n+2)}\left[720\lambda^{2}-120\lambda P^{2}(n^{2}+n-5)+P^{4}(5n^{4}+22n^{3}-29n^{2}-46n+120)+576P\Omega^{2}(n+1)(n^{2}+2n-40)\right].$$ (A40)

For the axisymmetric drop, *n* =0, there is the additional volume constraint. Therefore, the perturbed solution has a particular inhomogeneous solution proportional to the Lagrange multiplier *µ* in addition to the homogeneous solution such that

$$W(s)=w_{0}s^{\alpha }(1+w_{2}s^{2}+w_{4}s^{4}+\ldots )+\mu (d_{2}s^{2}+d_{4}s^{4}+d_{6}s^{6}+\ldots ).$$ (A41)

The coefficients *w*_2_ and *w*_4_ are as in Eq. (A39) and Eq. (A40) with *n* = 0 and the terms proportional to *μ* are given by

$$d_{2}=-1/4,\;d_{4}=(3\lambda +P^{2})/192,\;d_{6}=(10944P\Omega^{2}-P^{4}-45\lambda P^{2}-90\lambda^{2})/207360.$$ (A42)

For this case, the expansions for *R*(*s*) and *W*(*s*) are used to get the necessary series expansion for the volume of the drop

$$\begin{array}{l} V(s)=\int_{0}^{s}R(s)W(s)ds\\ \;=\mu [-s^{4}/16+(\lambda +P^{2})s^{6}/384+\ldots ]+w_{0}[s^{2}/2-(\lambda /4+P^{2}/6)s^{4}/4\\ \;+(30\lambda^{2}+45\lambda P^{2}+16P^{4}-1152P\Omega^{2})s^{6}/11520+\ldots ]. \end{array}$$ (A43)

For the axisymmetric isolated drop, the perturbed solution gets an additional inhomogeneous term due to the constant angular momentum constraint so that

$$W(s)=w_{0}s^{\alpha }(1+w_{2}s^{2}+w_{4}s^{4}+\ldots )+\mu (d_{2}s^{2}+d_{4}s^{4}+d_{6}s^{6}+\ldots )+\beta (b_{4}s^{4}\ldots ).$$ (A44)

Since the leading order of the additional term is found to be *O*(*s*^4^), retaining only one term is sufficient for this additional series, with

$$\beta b_{4}=\frac{\pi B\Omega^{2}}{2ℐ}.$$ (A45)

Here *B* is the integral term Eq. (A29) from the integro-differential Sturm-Liouville equation and the coefficients *w*_2_ and *w*_4_ remain as in Eq. (A39) and Eq. (A40) with *n* = 0.
